# Horizontal healthcare utilization inequity in patients with rare diseases in Korea

**DOI:** 10.1186/s12939-023-01903-9

**Published:** 2023-05-17

**Authors:** Daewon Kang, Sang-Eun Choi

**Affiliations:** College of Pharmacy, Korea University, 2511 Sejongro Sejong, South Korea

**Keywords:** Rare disease, Horizontal inequity index, Real-world data

## Abstract

**Background:**

Rare diseases (RDs) are difficult to diagnose and expensive to treat. Thus, the South Korean government has implemented several policies to help RD patients, including the Medical Expense Support Project, supporting low- to middle-income RD patients. However, no study in Korea has yet addressed health inequity in RD patients. This study assessed inequity trends in the medical utilization and expenditures of RD patients.

**Methods:**

This study measured the horizontal inequity index (HI) of RD patients and an age- and sex-matched control group using the National Health Insurance Service data from 2006 to 2018. Sex, age, number of chronic diseases, and disability variables were used to model expected medical needs and adjust the concentration index (CI) for medical utilization and expenditures.

**Results:**

The HI index of healthcare utilization in RD patients and the control group ranged from -0.0129 to 0.0145, increasing until 2012 and fluctuating since then. This increasing trend was more apparent for inpatient utilization in the RD patient group than in the outpatient group. The same index in the control group ranged from -0.0112 to -0.0040 without a significant trend. The healthcare expenditure HI in RD patients rose from -0.0640 to -0.0038, showing pro-poor values but moving toward a pro-rich state. In the control group, the HI for healthcare expenditures remained between 0.0029 and 0.0085.

**Conclusions:**

The HI of inpatient utilization and inpatient expenditures increased in a pro-rich state. The study results showed that implementing a policy that supports inpatient service utilization could help achieve health equity for RD patients.

**Supplementary Information:**

The online version contains supplementary material available at 10.1186/s12939-023-01903-9.

## Background

Rare diseases (RDs) are characterized by low prevalence and chronicity, and they profoundly affect the quality of life of patients and their families. The definition of RD varies across countries. For example, in the United States, a disease is categorized as an RD if it affects fewer than 200,000 people [1], whereas, in Europe, a disease can be classified as an RD if it affects fewer than 5 in every 10,000 people [2]. In Korea, the Rare Disease Management Act (2015) defined RD as a disease affecting fewer than or equal to 20,000 patients. In Korea, 55,499 people were newly diagnosed with an RD in 2019 [3], and the number is expected to increase as the government designates more RDs every year. Due to their rarity, the time and costs needed to diagnose and treat RDs are high. When it is difficult to obtain an accurate diagnosis and proper treatment for a disease, patients with lower incomes may experience more difficulties than those who are more affluent [4]. Diseases with these characteristics may exacerbate health and economic inequity. Therefore, if there is a structural reason for this inequity, the government should intervene for society’s sake.

The Ministry of Health and Welfare of Korea runs various social security programs, including National Health Insurance (approximately 96% of the population) and Medical Aid (approximately 4% of the population). Eligibility criteria for Medical Aid include age (under 18 or over 65), disability, refugees, and low income. The recipients of Medical Aid are people facing a severe economic challenge [5].

Korean patients can visit any medical institution without a referral from a primary care physician. They are inclined to go to large hospitals for high-quality care when the illness appears to be severe. [6]. Since 70 ~ 80% of the licensed doctors in Korea from 2010 to 2020 were specialists [7], it is reasonable to assume that most patients with serious illnesses visited specialists.

Along with the National Health Insurance Scheme, there are programs to help RD patients, including a copayment reduction system for incurable and RDs (1983 ~), which reduces out-of-pocket (OOP) payments to 10%; the Medical Expense Support Project (2001 ~), which covers OOP payments for low- to middle-income RD patients with general tax revenue; and Support for Catastrophic Health Expenditure (2001 ~). The registration of RD patients eligible for copayment reductions was implemented in July 2009, and the inpatient and outpatient OOP payment rate for registered patients was decreased from 20 to 10%. The OOP payment rate for high-cost imaging tests, including positron emission tomography (PET), computed tomography (CT), and magnetic resonance imaging (MRI), for diagnosing RDs was decreased from 50 to 10% for registered RD patients. The number of designated RDs eligible for these programs has increased, resulting in the expanded breadth and depth of healthcare coverage for RD patients. The RDs eligible for copayment reductions include diseases without a diagnostic process and KCD code, such as chromosomal abnormalities. Undiagnosed RDs are also eligible for copayment reductions through the undiagnosed disease program. The codes can be found on the helpline webpage (https://helpline.kdca.go.kr/cdchelp/).

Besides direct financial support, the Korean government has opened a Rare Disease Helpline website to provide information on RDs (2006 ~). The website provides information on each RD, including symptoms, causes, diagnosis, treatment, and disease codes for copayment reduction, the Medical Expense Support Project, educational content for patients and medical professionals, an online counseling service, and RD support centers.

The RD support centers are university hospitals where RD professionals share information on RDs, research new treatments, and train new professionals. For patients, the RD support centers provide an early diagnosis without performing redundant tests, genetics counseling, information on supportive programs, registration, and follow-up management. Four regional RD support centers were designated in 2006, and eight other institutions were added in 2019, including a central RD support center, which manages the RD center network.

Health equity studies conducted in South Korea showed that low-income patients tended to utilize medical services more than high-income patients because they had greater healthcare needs. Despite these differing needs, there was a dominant pro-rich trend for medical expenditures in the 2000s and 2010s [8–11]. During the same period, the horizontal inequity index (HI) for medical utilization indicated pro-poor or equal usage of healthcare services [8–11]. Although studies on healthcare utilization equity among the general population and cancer patients have been conducted, to our knowledge, no such study has been conducted on RD patients in South Korea. Given that policies have been implemented for RD patients, it is feasible to draw the implications of those policies by measuring the HI of medical utilization by RD patients and comparing the trend to that of a control group. Thus, to measure disparities in RD patients’ healthcare utilization depending on income level, we measured and assessed the trends in the HI for medical utilization and expenditures of RD patients using real-world data.

## Methods

### Study design and data

Korean National Health Insurance Service (NHIS) insurance claims data were used for this study. The NHIS provides customized cohort data upon request after reviewing the Institutional Review Board (IRB) approval and study protocol. Our data included the medical costs and socio-demographic information of 565,050 patients who had utilized medical services to treat RDs from 2006 to 2018, as well as 565,050 age and sex-matched controls over the same time period. We used the control group to compare HI trends and observe the impact of governmental policies introduced for RD patients. The controls were patients who had used medical services, including both general practitioner and specialist visits during the same period for illnesses other than RDs. We conducted a nested case‐control study in which patients with records of medical utilization for RD treatment each year were defined as RD patients, and the control group was randomly assigned after age and sex matching. To analyze inequities in healthcare utilization and healthcare expenditures per year, we measured the HI suggested by Wagstaff and Van Doorslaer [12] and compared trends.

### Variables

There were six dependent variables for measuring HI: total medical utilization, inpatient utilization, outpatient utilization, total medical expenditure, inpatient expenditure, and outpatient expenditure. Medical utilization, inpatient utilization, and outpatient utilization were defined as the number of records in the data for total hospital visits (inpatient visits and outpatient visits combined), inpatient visits, and outpatient visits for each year, respectively. Medical expenditure, inpatient expenditure, and outpatient expenditure were defined as the sum of the data’s total medical costs, inpatient costs, and outpatient costs for each year, respectively.

Subjects must have income level data to obtain the concentration index (CI) [13–15]. The NHIS data has an insurance premium amount variable, which can serve as a proxy index for income class and enable CI extrapolation because it reflects patients’ income and property for each year [16]. The data includes patients who pay no insurance premiums because they are eligible for Medical Aid.

To estimate expected medical needs, we used independent variables, including age group, sex, disability, and the number of chronic diseases that are not RDs [17–19]. Age groups were divided into 10 categories as dummy variables (0 – 1, 2 – 10, 11 – 20, 21 – 30, 31 – 40, 41 – 50, 51 – 60, 61 – 70, 71 – 80, and over 80), rather than treating age as a continuous variable, because each age group might have different medical needs [10]. Whether a patient had a disability was also used as a dummy variable. The number of chronic diseases was counted if a patient had a disease considered a chronic disease on the NHIS disease statistics service with the following International Classification of Diseases (ICD) codes: tuberculosis (A15-16, and A19), chronic viral infections (B18, B19, and K70-K77), neoplasms (C00-C97, and D00-D09), disorders of the thyroid gland (E00-E07), diabetes mellitus (E10-E14), mental and behavioral disorders (F00-F99), diseases of the nervous system (G00-G83), diseases of the circulatory system (I05-I15, I20-I27, I30-I52, and I60-I69), and chronic kidney disease (N18) [20].

### Measuring healthcare utilization and expenditure equity

Unlike the CI, the HI assumes that different individuals have different medical needs depending on their socio-economic status, and equity is achieved when patients with greater medical needs utilize more medical services accordingly [21–23]. The HI can have a value between -1 and 1. An HI value close to -1 indicated that patients with low income utilized most medical services. If the HI value was 0, one could assume that medical services were equally utilized among different income groups. The HI value only provides the direction and relative degree of inequity. The HI can be calculated by adjusting the CI of actual measurement (Cm) with the CI of estimated medical needs (Cn) using the following equation [24].

1 $$\mathrm{HI }=\mathrm{ Cm}-\mathrm{Cn}$$

To obtain the Cn, the patients’ expected medical expenditures and utilization for each year were needed. We used a generalized linear model (GLM) with the variables described above to estimate the expected medical needs for computing the HI [25].

The following equation developed by Wagstaff was used to measure the CI of health expenditures and frequency of medical utilization [26].

2 $$C=\frac{2}{\mu }cov({y}_{i},{R}_{i})$$

where C is the CI, μ is the mean of the dependent health variable, and cov (y_*i*_, R_*i*_) is the covariance of the health variable of the *i*th individual, y_*i*_, and the fractional income level rank of the *i*th individual, R_*i*_. We calculated the HI of both the RD patients and control groups from 2006 to 2018 and compared trends using the SAS 9.4 statistics package and Microsoft Excel 2020.

## Results

### Data characteristics

The subjects’ characteristics are presented in Table 1. After matching, the data had 141,493 matches in 2006 and 296,846 in 2018. The mean age increased from 42.5 years in 2006 to 48.8 in 2018. There was a significant difference every year in the distribution of income level, disability ratio, and the number of chronic diseases between the RD group and the control group. The RD group had more persons eligible for Medical Aid and patients with the highest income than the control group. The RD group also had more disabled patients than the control group (20.1% – 24.1% vs. 5.7 – 7.1%). The average number of chronic conditions was higher in RD patients than in the control group (2.74 – 3.46 vs. 1.14 – 1.82).

**Table 1:** Characteristics of the subjects

	2006		2009		2012		2015		2018	
Group	control	case	control	case	control	case	control	case	control	case
N	141,493	141,493	187,269	187,269	209,415	209,415	251,971	251,971	296,846	296,846
Male %	55	55	54	54	54	54	53	53	53	53
Mean age	42.5	42.5	44.4	44.4	43.4	43.4	46.9	46.9	48.8	48.8
Age group (%)										
0–1	2.1	2.1	1.9	1.9	1.7	1.7	1.5	1.5	1.0	1.0
2–10	8.7	8.7	7.3	7.3	7.0	7.0	6.6	6.6	6.2	6.2
11–20	9.0	9.0	9.3	9.3	9.3	9.3	8.4	8.4	7.3	7.3
21–30	10.2	10.2	9.5	9.5	8.8	8.8	8.8	8.8	8.9	8.9
31–40	13.4	13.4	12.1	12.1	11.3	11.3	10.3	10.3	9.6	9.6
41–50	17.0	17.0	16.0	16.0	14.9	14.9	14.3	14.3	13.5	13.5
51–60	15.5	15.5	16.6	16.6	18.6	18.6	18.9	18.9	18.3	18.3
61–70	14.4	14.4	15.2	15.2	14.8	14.8	15.4	15.4	17.5	17.5
71–80	8.1	8.1	9.9	9.9	10.8	10.8	12.2	12.2	12.9	12.9
> 80	1.6	1.6	2.2	2.2	2.6	2.6	3.6	3.6	4.7	4.7
Income level (%)^*^										
0^**^	4.35	10.04	4.5	9.26	3.89	8.41	3.48	8.22	3.63	7.88
1	8.67	7.27	6.86	6.75	7.23	7.44	7.31	7.62	7.41	7.7
2	5.42	4.64	6.44	5.43	6.61	5.63	7.8	6.95	7.97	7.16
3	6.21	5.17	7.01	5.98	7.43	6.48	6.05	5.3	5.99	5.31
4	8.69	7.61	7.11	6.25	6.97	6.14	7.31	6.46	7.43	6.69
5	8.2	7.36	8.33	7.33	8.28	7.47	8.16	7.21	7.98	7.22
6	9.07	8.21	9.43	8.6	9.02	8.15	9.03	8.17	8.84	8.14
7	9.85	8.95	10.43	9.69	10.18	9.45	10.24	9.61	10.08	9.41
8	13.26	12.61	11.99	11.43	11.76	11.3	11.79	11.23	11.65	11.14
9	12.87	13.13	13.56	13.61	13.66	13.52	13.61	13.51	13.67	13.53
10	13.42	15	14.36	15.67	14.96	16	15.23	15.74	15.35	15.81
Disabled (%)^*^	5.7	20.1	7.1	24.1	7.1	24.1	6.9	23.8	7.1	23.5
Chronic condition (N)^*^	1.14	2.74	1.32	2.79	1.46	2.97	1.60	3.25	1.82	3.46

^*^p < 0.05

^**^persons eligible for Medical Aid

### Concentration index and horizontal inequity index of the subjects

**Fig. 1:**
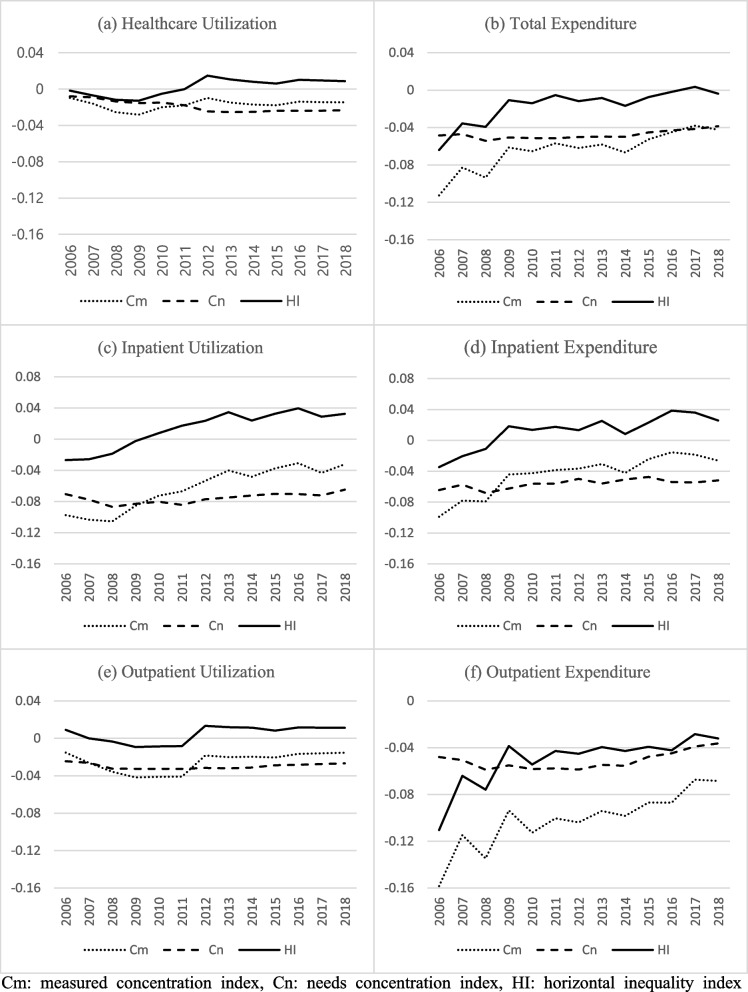
Concentration indices of rare disease patients

The Cn, Cm, and HI of healthcare utilization, inpatient utilization, outpatient utilization, total expenditures, inpatient expenditures, and outpatient expenditures of RD patients are presented in Fig. 1. For RD patients, the healthcare utilization Cm decreased until 2009 (-0.0097 to -0.0282) and rose to -0.0099 in 2012. After 2012, the Cm level trended at a similar level. Values below 0 over the study period indicated that patients with low incomes utilized healthcare services more than patients with high incomes (pro-poor). The Cn of total healthcare utilization decreased, indicating that the needs of poorer patients for medical care had increased. The Cm and Cn values of total healthcare utilization have elevated the HI value above 0 since 2011. This means that considering the medical needs of the RD patients by income level, patients with higher incomes utilized medical services more than those with lower incomes. The Cm for RD inpatient utilization steadily increased (-0.0974 to -0.0261), and the Cn stayed in a range between -0.0868 and -0.0648. The HI followed the trend of the Cm as a positive value from 2010. Outpatient utilization Cm values stayed below 0 throughout the entire period, with a significant increase in 2012 (-0.0474 to -0.0154). After adjusting with the Cn, the outpatient utilization HI values became close to 0 (-0.0095 to 0.0119). When the values were compared, the outpatient utilization HI represented less inequity in any direction than the inpatient utilization HI.

The total healthcare expenditure Cm of RD patients increased from -0.1126 to -0.0424. Over the same period, Cn values remained similar (-0.0542 to 0.0386), and HI came close to 0, representing near-equity in healthcare expenditures since 2009 (-0.0640 to 0.0035). The Cm values of inpatient expenditures were generally higher than the Cm values of total expenditures (-0.0989 to 0.0154), while the Cn values of inpatient expenditures were lower than those of total expenditures (-0.0680 to -0.0473). The HI of RD patients’ inpatient expenditures followed the trend in Cm (-0.0422 ~ 0.0386), with values above 0 (pro-rich) since 2009. The outpatient expenditure Cm measures were lower than other expenditure indexes and increased over time (-0.1583 to -0.067). The Cn of outpatient expenditures trended within a range of -0.0587 to -0.0363. The HI of the outpatient expenditure stayed below 0 throughout the entire period (-0.1104 to -0.0284). The HI values of medical expenditures indicated that patients with higher incomes utilized more inpatient services than those with lower incomes. The complete index data of RD patients can be found in Supplementary Table 1.

The Cn, Cm, and HI of healthcare utilization, inpatient utilization, outpatient utilization, total expenditures, inpatient expenditures, and outpatient expenditures of the control group are presented in Supplementary Table 2. All of the control group indexes showed less fluctuation than those of the RD patients. The Cm, Cn, and HI values for total healthcare utilization ranged between -0.0183 and -0.0080, -0.0117 and -0.0017, and -0.0112 and -0.004, respectively. The inpatient utilization Cm and Cn values were close to each other, resulting in near-zero HI values (-0.0326 to -0.0264, -0.0304 to -0.0255, and -0.0067 to -0.00002, respectively). The Cm and Cn values for outpatient utilization in the control group were also similar, and the HI values were close to 0 (-0.0146 to -0.0044, -0.0159 to -0.0067, and -0.0053 to -0.01156, respectively). The Cm values for total medical expenditures in the control group were negative values that were higher than the Cn values, resulting in positive HI values over the entire period (-0.0221 to -0.0144, -0.0287 to -0.0228, and 0.0029 to 0.0085, respectively). The trends in Cm, Cn, and HI values were similar to inpatient expenditures. Nevertheless, the gap between Cm and Cn values was slightly larger, resulting in slightly higher HI values (-0.0309 to -0.0243, -0.0208 to -0.0120, and 0.0070 to 0.0126, respectively).

### Trend comparison

**Fig. 2:**
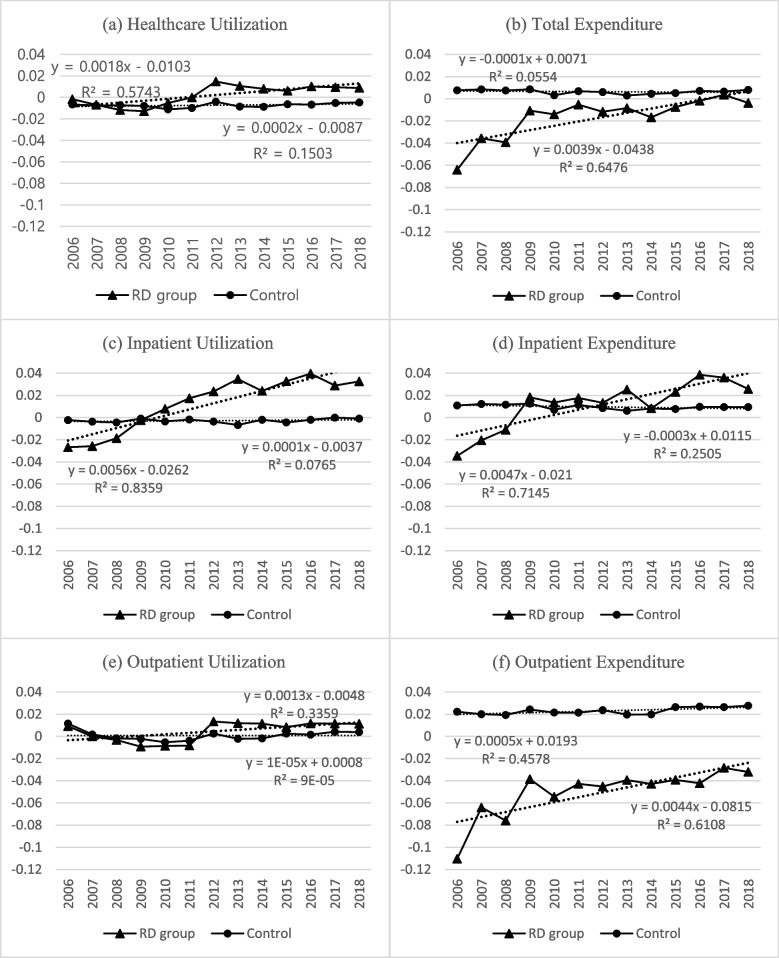
Horizontal inequity indices of rare disease patients and control group

HI trends in the RD and control group are presented in Fig. 2. RD patients exhibited a more apparent upward trend in healthcare utilization HI compared to the control group, especially in inpatient utilization. The upward trend was also evident in the expenditure HI. The trend line coefficients were higher in the healthcare expenditure HI than the utilization HI, with the exception of inpatient utilization.

## Discussion

This study computed the HI of six dependent variables (total medical utilization, inpatient utilization, outpatient utilization, total medical expenditures, inpatient expenditures, and outpatient expenditures) of RD patients and a control group from 2006 to 2018.

The HI for the control group (representing the general population without RD) showed steady values for all dependent variables. In outpatient and inpatient utilization, the measured and needs-expected CI values were similar. They resulted in near-zero values, meaning that patients from different economic backgrounds utilized medical services according to their needs, not favoring any particular group. Unlike medical utilization, the HI measures for medical expenditures had positive values after adjusting for the needs-expected CI, indicating that high-income patients utilized more high-quality services, especially in outpatient expenditures. The results from the control group were consistent with previous studies [8–11].

Regarding the RD group, the needs-expected CI values for outpatient expenditures were higher than the measured CI values during the study period, and all CI values were below 0. These results mean that low-income patients spent more money than expected, which could be an effect of the Medical Expense Support Project, which made medical costs for low- to middle-income class RD patients free. All six HI measures of the RD group showed upward trends. The HI measures for inpatient utilization and inpatient expenditures of RD patients were higher than those of the control group and were 0 since 2009. This means that high-income RD patients have used inpatient healthcare services more since 2009 than low-income RD patients, and this pro-rich trend has become stronger over time. This change can be partially attributable to the RD patient registration policy implemented in 2009 because it helps patients with incomes above 120% of the median income and hence, are ineligible for the Medical Expense Support Project to utilize high-cost inpatient medical care by reducing the OOP payment rate. A health equity study on RD patients conducted in China revealed a positive correlation between medical expenses related to RD and higher income, as well as a higher reimbursement ratio [27]. A pro-rich trend was also evident in the medical treatment of cancer patients in Korea, consistent with the findings of this study. This was further supported by the fact that copayment reductions were also applied to cancer treatment. [28, 29]

Although HI values for outpatient expenditures stayed negative, an upward trend was apparent. If the trend continues, it will generate pro-rich values in a few years. The upward trend in the HI for outpatient utilization was relatively insignificant, judging from the coefficient of the trend line.

It is clear that HI trends for RD patients differed from the control group, which could be due to the characteristics of RDs and policies implemented for RD patients. For instance, the expansive new technologies and medicines for RD might account for the upward trend in the HI. The HI for outpatient expenditures might be lower than zero due to the Medical Expense Support Project because it covers OOP payments for low- to middle-income patients [30]. Persons eligible for the project are RD patients with less than 120% of the median household income in Korea each year. Patients with high-cost RDs, including Gaucher’s disease, Fabry’s disease, hemophilia, and mucopolysaccharidosis (MPS), would be eligible for the project if they had incomes less than 160% of the median income. This project might play an important role in reducing the disparity in healthcare service utilization among RD patients. According to a survey conducted in 2018, 62.64% of RD patients said that expanding financial support should be prioritized in helping RD patients [31]. While all of these programs can help RD patients, the HI is moving toward a pro-rich state, especially in inpatient utilization and expenditures. The OOP payment burden involved in inpatient services and non-reimbursable medical services following inpatient services can be the reason for this trend. According to the survey in 2018, 58.65% of RD patients said non-reimbursable medical costs were a heavy burden. Also, 24.22% of the patients said there were unmet medical needs for RDs because they could not afford them [31].

The HI can be difficult to interpret [32] and compare with other study results because of different data, data handling methods, variables, and study periods. We tried to overcome this problem by measuring the index over multiple years and using a control group of non-RD patients from the same data. The HI of the control group, with results patterns similar to those of previous studies, can increase the reliability of the results and render HI comparisons robust. Kim (2018) used this method to compare the HI of medical utilization and expenditures by types of diseases (general vs. severe vs. chronic) [9]. However, there still were limitations due to the data set and the rarity of the target diseases. First of all, the NHIS data does not cover non-reimbursable services, which can be a significant part of the burden for patients [33]. A survey of OOP costs for RD patients by income level would help overcome this limitation. Second, not all the records reflect reality because service providers can alter disease codes for reimbursement [34]. Also, the estimation of the expected need might be limited because the data do not provide factors related to health-related behavior, including education level, marital status, drinking or smoking habits, and the usage of over-the-counter (OTC) drugs [35]. Another limitation is that it does not consider the characteristics of each RD, such as seriousness or anatomical site, because too many RDs are involved, and many of them have no known natural history. Nevertheless, this was the first study on the medical utilization equity of RD patients using real-world data compared to non-RD patients over a period of multiple years. Therefore, the results of this study can be used as evidence for implementing policies to support inpatient healthcare services for low-income RD patients.

## Conclusions

The HI of inpatient utilization and expenditures indicated a pro-rich state since 2009, and the trend was strengthening. The outpatient utilization HI after 2011 showed more of a pro-rich state than the control group. The HI for outpatient expenditures was pro-poor throughout the study period but moving toward a pro-rich state. Thus, in general, the HI measures are moving toward a pro-rich state for RD patients, while those of the control group were steady. Analyzing the HI trend over an extended period after 2013 could confirm whether the index has plateaued.

Further equity studies that take into account non-reimbursable medical care and nursing care expenses can provide a more comprehensive understanding of healthcare utilization and equity in access by patients with rare diseases. Additionally, more specific equity studies could be conducted by categorizing individual rare diseases according to their chronicity, severity, or availability of treatment. To reduce healthcare inequity among patients with rare diseases, the government could consider either adjusting eligibility criteria for the Medical Expense Support Project to include more patients or reducing the out-of-pocket payment ratio for insured medical services to 5% for the first five years after diagnosis.

## Supplementary Information

**Additional file 1: Table S1. **Concentration index for expected medical need (Cn), Concentration index for measured medical need (Cm), Horizontal inequity index (HI) in rare disease patients.** Table S2.**Concentration index for expected medical need (Cn), Concentration index for measured medical need (Cm), and Horizontal inequity index (HI) in rare disease patients.

## Data Availability

The original dataset has been removed from the NHIS server since the time of writing. The analyzed data are available from the main author upon reasonable request.

## Abbreviations

CI: Concentration index
CC: Concentration curve
Cn: Concentration index of expected needs
Cm: Measured concentration index
HI: Horizontal inequity index
RD: Rare disease
