# Supplementary material for: Horizontal healthcare utilization inequity in patients with rare diseases in Korea
Source: Int J Equity Health. 2023 May 17;22:93. doi: 10.1186/s12939-023-01903-9 (PMC10190067; doi:10.1186/s12939-023-01903-9)
Supplement: Supplementary file 1 — Additional file 1: Table S1. Concentration index for expected medical need (Cn), Concentration index for measured medical need (Cm), Horizontal inequity index (HI) in rare disease patients. Table S2.Concentration index for expected medical need (Cn), Concentration index for measured medical need (Cm), and Horizontal inequity index (HI) in rare disease patients. [file 12939_2023_1903_MOESM1_ESM.docx]

| Index | | 2006 | 2007 | 2008 | 2009 | 2010 | 2011 | 2012 | 2013 | 2014 | 2015 | 2016 | 2017 | 2018 |
| --- | --- | --- | --- | --- | --- | --- | --- | --- | --- | --- | --- | --- | --- | --- |
| Total Utilization | Cm | -0.0097 | -0.0161 | -0.0255 | -0.0282 | -0.0201 | -0.0179 | -0.0099 | -0.0149 | -0.0171 | -0.0178 | -0.0139 | -0.0145 | -0.0145 |
|  | Cn | -0.0080 | -0.0091 | -0.0137 | -0.0154 | -0.0148 | -0.0178 | -0.0246 | -0.0254 | -0.0251 | -0.0238 | -0.0240 | -0.0239 | -0.0233 |
|  | HI | -0.0017 | -0.0070 | -0.0118 | -0.0129 | -0.0053 | -0.0001 | 0.0147 | 0.0105 | 0.0080 | 0.0060 | 0.0101 | 0.0094 | 0.0087 |
| Inpatient Utilization | Cm | -0.0974 | -0.1033 | -0.1054 | -0.0854 | -0.0725 | -0.0669 | -0.0535 | -0.0401 | -0.0483 | -0.0374 | -0.0308 | -0.0433 | -0.0322 |
|  | Cn | -0.0706 | -0.0775 | -0.0868 | -0.0830 | -0.0802 | -0.0842 | -0.0770 | -0.0748 | -0.0723 | -0.0701 | -0.0704 | -0.0722 | -0.0648 |
|  | HI | -0.0268 | -0.0258 | -0.0186 | -0.0024 | 0.0078 | 0.0173 | 0.0235 | 0.0347 | 0.0240 | 0.0327 | 0.0396 | 0.0288 | 0.0326 |
| Outpatient Utilization | Cm | -0.0153 | -0.0262 | -0.0357 | -0.0417 | -0.0411 | -0.0409 | -0.0183 | -0.0201 | -0.0197 | -0.0204 | -0.0166 | -0.0159 | -0.0154 |
|  | Cn | -0.0244 | -0.0262 | -0.0324 | -0.0325 | -0.0325 | -0.0327 | -0.0316 | -0.0320 | -0.0312 | -0.0287 | -0.0282 | -0.0274 | -0.0304 |
|  | HI | 0.0091 | 0.0000 | -0.0033 | -0.0092 | -0.0086 | -0.0083 | 0.0134 | 0.0119 | 0.0115 | 0.0083 | 0.0117 | 0.0115 | 0.0113 |
| Total Expenditure | Cm | -0.1126 | -0.0827 | -0.0935 | -0.0614 | -0.0654 | -0.0568 | -0.0619 | -0.0581 | -0.0667 | -0.0527 | -0.0449 | -0.0381 | -0.0424 |
|  | Cn | -0.0485 | -0.0471 | -0.0542 | -0.0506 | -0.0513 | -0.0515 | -0.0501 | -0.0497 | -0.0500 | -0.0452 | -0.0431 | -0.0416 | -0.0386 |
|  | HI | -0.0640 | -0.0356 | -0.0393 | -0.0107 | -0.0141 | -0.0053 | -0.0117 | -0.0084 | -0.0168 | -0.0075 | -0.0019 | 0.0035 | -0.0038 |
| Inpatient Expenditure | Cm | -0.0989 | -0.0779 | -0.0791 | -0.0441 | -0.0425 | -0.0384 | -0.0366 | -0.0306 | -0.0422 | -0.0243 | -0.0154 | -0.0184 | -0.0261 |
|  | Cn | -0.0643 | -0.0574 | -0.0680 | -0.0624 | -0.0561 | -0.0560 | -0.0499 | -0.0559 | -0.0505 | -0.0473 | -0.0540 | -0.0545 | -0.0519 |
|  | HI | -0.0346 | -0.0205 | -0.0111 | 0.0183 | 0.0136 | 0.0176 | 0.0133 | 0.0253 | 0.0083 | 0.0231 | 0.0386 | 0.0360 | 0.0257 |
| Outpatient Expenditure | Cm | -0.1583 | -0.1147 | -0.1347 | -0.0936 | -0.1126 | -0.1004 | -0.1039 | -0.0941 | -0.0983 | -0.0869 | -0.0870 | -0.0673 | -0.0684 |
|  | Cn | -0.0643 | -0.0574 | -0.0680 | -0.0624 | -0.0561 | -0.0560 | -0.0499 | -0.0559 | -0.0505 | -0.0473 | -0.0540 | -0.0545 | -0.0519 |
|  | HI | -0.1104 | -0.0641 | -0.0759 | -0.0386 | -0.0544 | -0.0428 | -0.0452 | -0.0394 | -0.0428 | -0.0392 | -0.0422 | -0.0284 | -0.0321 |

**Table 1. Concentration index for expected medical need (Cn), Concentration index for measured medical need (Cm), Horizontal inequity index (HI) in rare disease patients**

| Index | | 2006 | 2007 | 2008 | 2009 | 2010 | 2011 | 2012 | 2013 | 2014 | 2015 | 2016 | 2017 | 2018 |
| --- | --- | --- | --- | --- | --- | --- | --- | --- | --- | --- | --- | --- | --- | --- |
| Total Utilization | Cm | -0.0183 | -0.0133 | -0.0140 | -0.0173 | -0.0143 | -0.0152 | -0.0080 | -0.0115 | -0.0105 | -0.0117 | -0.0098 | -0.0129 | -0.0118 |
|  | Cn | -0.0117 | -0.0057 | -0.0066 | -0.0093 | -0.0031 | -0.0054 | -0.0040 | -0.0028 | -0.0017 | -0.0053 | -0.0031 | -0.0077 | -0.0071 |
|  | HI | -0.0066 | -0.0076 | -0.0074 | -0.0080 | -0.0112 | -0.0099 | -0.0040 | -0.0086 | -0.0088 | -0.0064 | -0.0067 | -0.0052 | -0.0047 |
| Inpatient Utilization | Cm | -0.0326 | -0.0292 | -0.0307 | -0.0319 | -0.0323 | -0.0300 | -0.0317 | -0.0325 | -0.0284 | -0.0316 | -0.0285 | -0.0277 | -0.0264 |
|  | Cn | -0.0303 | -0.0255 | -0.0263 | -0.0310 | -0.0288 | -0.0283 | -0.0280 | -0.0258 | -0.0264 | -0.0272 | -0.0265 | -0.0276 | -0.0255 |
|  | HI | -0.0022 | -0.0037 | -0.0045 | -0.0009 | -0.0035 | -0.0018 | -0.0036 | -0.0067 | -0.0020 | -0.0044 | -0.0020 | 0.0000 | -0.0009 |
| Outpatient Utilization | Cm | -0.0044 | -0.0076 | -0.0113 | -0.0146 | -0.0120 | -0.0131 | -0.0060 | -0.0094 | -0.0078 | -0.0068 | -0.0057 | -0.0077 | -0.0075 |
|  | Cn | -0.0159 | -0.0093 | -0.0096 | -0.0123 | -0.0067 | -0.0092 | -0.0086 | -0.0072 | -0.0060 | -0.0093 | -0.0073 | -0.0119 | -0.0114 |
|  | HI | 0.0116 | 0.0018 | -0.0018 | -0.0022 | -0.0053 | -0.0039 | 0.0025 | -0.0022 | -0.0019 | 0.0025 | 0.0016 | 0.0042 | 0.0038 |
| Total Expenditure | Cm | -0.0195 | -0.0143 | -0.0163 | -0.0202 | -0.0221 | -0.0203 | -0.0199 | -0.0211 | -0.0189 | -0.0210 | -0.0186 | -0.0207 | -0.0175 |
|  | Cn | -0.0271 | -0.0228 | -0.0238 | -0.0287 | -0.0253 | -0.0271 | -0.0260 | -0.0241 | -0.0233 | -0.0261 | -0.0257 | -0.0272 | -0.0255 |
|  | HI | 0.0076 | 0.0084 | 0.0074 | 0.0085 | 0.0032 | 0.0068 | 0.0060 | 0.0029 | 0.0044 | 0.0051 | 0.0071 | 0.0065 | 0.0080 |
| Inpatient Expenditure | Cm | -0.0157 | -0.0120 | -0.0134 | -0.0183 | -0.0207 | -0.0173 | -0.0195 | -0.0197 | -0.0169 | -0.0193 | -0.0172 | -0.0178 | -0.0159 |
|  | Cn | -0.0266 | -0.0243 | -0.0251 | -0.0309 | -0.0278 | -0.0288 | -0.0280 | -0.0258 | -0.0250 | -0.0270 | -0.0269 | -0.0273 | -0.0253 |
|  | HI | 0.0109 | 0.0122 | 0.0116 | 0.0126 | 0.0070 | 0.0114 | 0.0085 | 0.0061 | 0.0081 | 0.0077 | 0.0096 | 0.0095 | 0.0094 |
| Outpatient Expenditure | Cm | -0.0118 | -0.0087 | -0.0094 | -0.0080 | -0.0080 | -0.0106 | -0.0067 | -0.0090 | -0.0076 | -0.0044 | -0.0031 | -0.0062 | -0.0036 |
|  | Cn | -0.0340 | -0.0287 | -0.0286 | -0.0323 | -0.0295 | -0.0320 | -0.0303 | -0.0286 | -0.0274 | -0.0308 | -0.0300 | -0.0326 | -0.0313 |
|  | HI | 0.0222 | 0.0200 | 0.0192 | 0.0243 | 0.0215 | 0.0214 | 0.0237 | 0.0196 | 0.0198 | 0.0264 | 0.0268 | 0.0264 | 0.0277 |

**Table 2. Concentration index for expected medical need (Cn), Concentration index for measured medical need (Cm), and Horizontal inequity index (HI) in rare disease patients**
